# An atypical case of Kawasaki disease with severe pneumonia in a neonate

**DOI:** 10.1186/s12887-022-03203-7

**Published:** 2022-03-14

**Authors:** Yoshiki Kawamura, Hiroki Miura, Kazuyoshi Saito, Takayuki Kanno, Tadafumi Yokoyama, Yuta Aizawa, Tetsushi Yoshikawa

**Affiliations:** 1 Department of Pediatrics, Fujita Health University School of Medicine, 1-98 Kutsukake-cho, Dengakugakubo, Toyoake, Aichi 47-01192 Japan; 2 Department of Pathology, National Institute of Infectious Diseases, 1-23-1 Toyama, Shinjuku-ku, Tokyo, 162-8640 Japan; 3 Department of Pediatrics, School of Medicine, Institute of Medical, Pharmaceutical and Health Sciences, Kanazawa University, 13-1 Takara-machi, Kanazawa, Ishikawa 920-8640 Japan; 4 Department of Pediatrics, Niigata University Graduate School of Medical and Dental Sciences, 1-757 Asahimachi-Dori, Chuo-ku, Niigata, Niigata 951-8510 Japan

**Keywords:** Kawasaki disease, Neonate, Pneumonia

## Abstract

**Background:**

Kawasaki disease (KD) is an acute, febrile, systemic vasculitis of unknown etiology that primarily affects the coronary arteries and generally occurs at around 1 year of age. Although the diagnosis of KD is generally not difficult, it is challenging in cases of incomplete KD lacking characteristic clinical manifestations. The incidence of incomplete KD is higher in infants younger than 6 months of age. Pneumonia is an extremely rare complication of KD and can be misinterpreted as atypical pneumonia rather than KD. Herein, we report a neonate with atypical KD and severe pneumonia who required mechanical ventilation.

**Case presentation:**

Japanese one-month-old infant had only fever and rash on admission (day 1), and he was transferred to the intensive care unit for severe pneumonia on day 2. Although pneumonia improved following intensive care, he was diagnosed with KD on day 14 because of emerging typical clinical manifestations such as fever, bulbar nonexudative conjunctival injection, desquamation of the fingers, and coronary artery aneurysm. KD symptoms improved after three doses of intravenous immunoglobulin plus cyclosporine. However, small coronary aneurysms were present at the time of discharge. In a retrospective analysis, no pathogens were detected by multiplex real-time PCR in samples collected at admission, and the serum cytokine profile demonstrated prominent elevation of IL-6 as well as elevation of neopterin, sTNF-RI, and sTNF-RII, which suggested KD.

**Conclusions:**

The patient’s entire clinical course, including the severe pneumonia, was caused by KD. As in this case, neonatal KD may exhibit atypical manifestations such as severe pneumonia requiring mechanical ventilation.

## Background

Kawasaki disease (KD) is an acute febrile systemic vasculitis with unknown etiology that primarily affects the coronary arteries. The incidence rate of the disease is high in East Asian countries, including Japan, and it most commonly occurs in children around 1 year of age [1]. The diagnostic criteria for KD are fever, bilateral bulbar conjunctival injection, changes in the lips and oral cavity, rash, changes in the peripheral extremities, and non-suppurative cervical lymphadenopathy [2]. Patients are diagnosed with typical KD if they fulfill at least five of these criteria or if they have four criteria and also demonstrate coronary artery aneurysms (CAA) on echocardiography.

Although it is not difficult to identify typical KD, the diagnosis of incomplete KD that lacks several clinical manifestations is challenging. The incidence of incomplete KD has been suggested to be higher in infants younger than 6 months of age [3]. Difficulty diagnosing incomplete KD results in delayed treatment, which may lead to the development of CAA [4]. Therefore, early diagnosis and initiation of intravenous immunoglobulin (IVIG) therapy are essential to reduce the risk of CAA in these patients.

Pneumonia is an extremely rare complication of KD [5–8], and can be misinterpreted as atypical pneumonia. Herein, we report a neonatal case of KD with severe pneumonia that required mechanical ventilation. The patient was admitted to the intensive care unit (ICU) for the treatment of severe pneumonia, but since the typical clinical features of KD only became apparent after ICU discharge, the diagnosis and treatment of KD were delayed.

### Case presentation

**Fig. 1:**
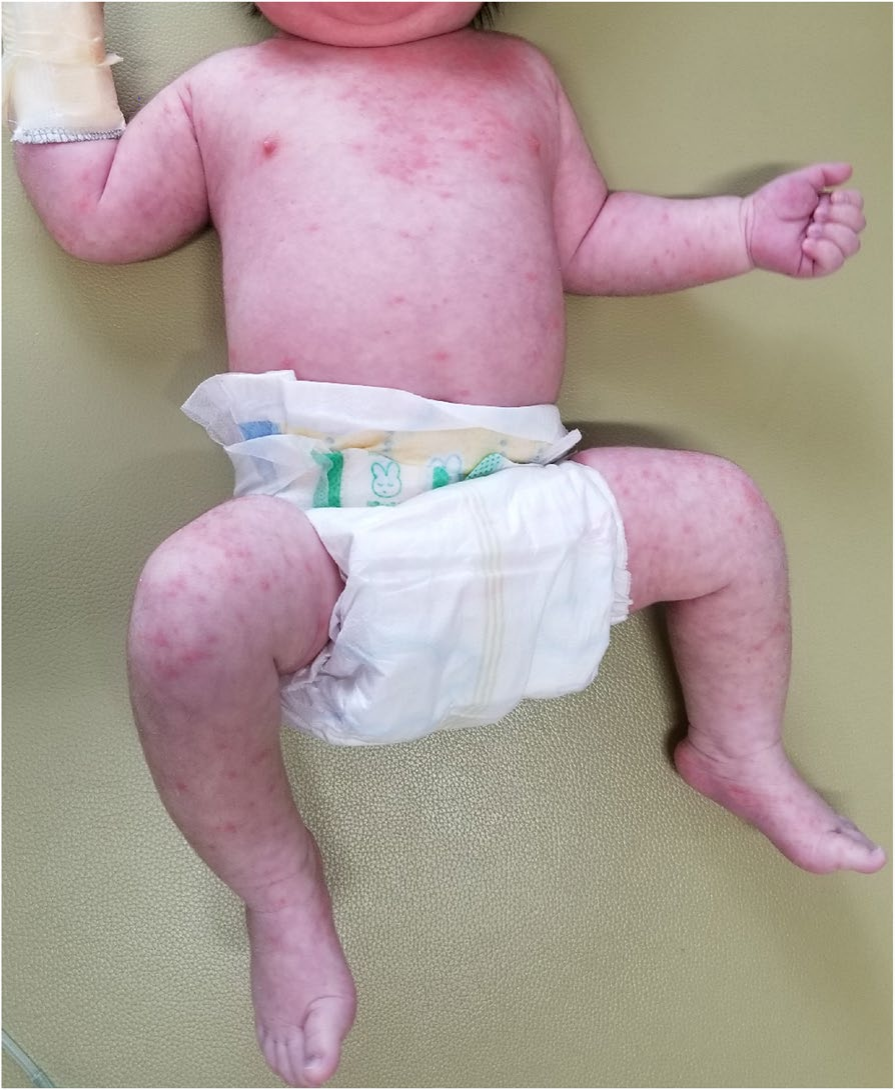
The photo shows erythematous, maculopapular eruptions over whole-body on day 1

A previously healthy 1-month-old boy was admitted to our university hospital with 1 day of fever (39.0 ℃), rhinorrhea, cough, and erythematous papules covering his whole body (Fig. 1). At the time of admission (day 1) he had no other symptoms suggestive of KD, such as extremity changes, conjunctivitis, oral changes, and cervical lymphadenopathy. Initial vital signs and laboratory results were as follows: blood pressure, 90/48 mmHg; heart rate, 142/min; respiratory rate (RR), 40/min; white blood cells (WBC), 10.2 × 10^3^/μL with neutrophil predominance; C-reactive protein (CRP), 32 mg/L; serum sodium, 135 mmol/L; serum albumin, 36 g/L; and serum aspartate/alanine aminotransferase, 22/13 units/L. Urinalysis and cerebrospinal fluid examination revealed no abnormal findings. Rapid diagnostic tests for respiratory syncytial virus, metapneumovirus, and *Mycoplasma pneumoniae* were negative, and severe acute respiratory syndrome coronavirus 2 was not detected by RT-PCR. The patient was treated with 150 mg/kg/day of cefotaxime, 60 mg/kg/day of vancomycin, and 60 mg/kg/day of acyclovir.

**Fig. 2:**
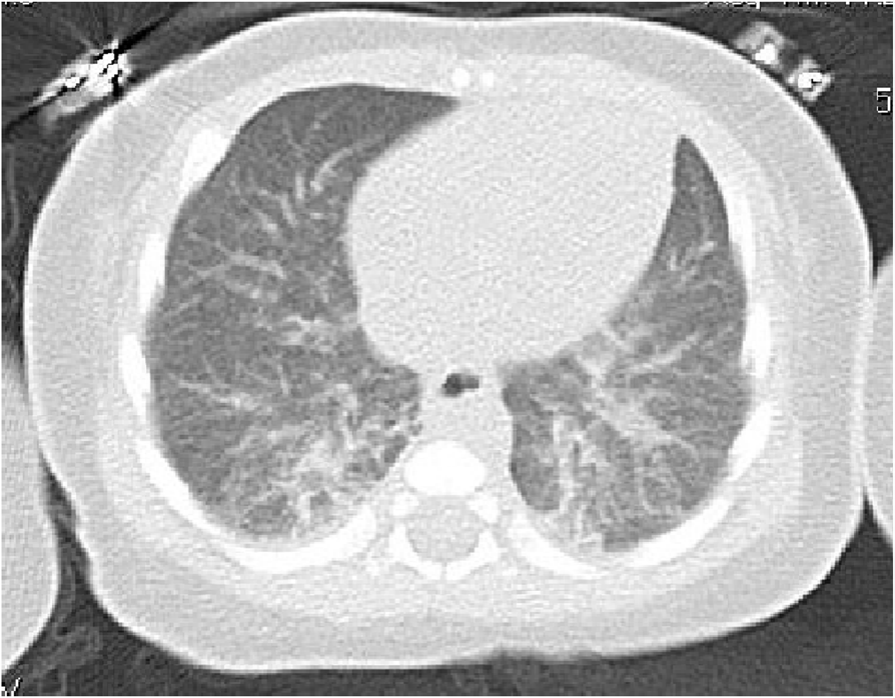
Chest CT demonstrated bilateral ground-glass infiltrates

On day 2 he exhibited frequent apnea (RR, 72/min), and blood gas analysis revealed hypercapnia (62.7 mmHg). Chest computerized tomography (CT) revealed bilateral consolidations (Fig. 2). He was transferred to the ICU and underwent mechanical ventilation. IVIG (500 mg/kg/day) was administered for 3 days as adjunctive treatment for severe infection [9]. He became afebrile on day 3, and acyclovir was discontinued due to negative PCR for herpes simplex virus. Moreover, administration of cefotaxime and vancomycin was discontinued due to negative blood and tracheal aspirate cultures on day 7. His condition gradually recovered without high fever or any clinical features suggesting KD, but elevated levels of CRP continued (63 mg/L on day 9). He was extubated on day 10 and discharged from the ICU on day 13. On day 14, however, fever recurred, and he also developed bilateral bulbar nonexudative conjunctival injection and desquamation of his fingers. KD was finally diagnosed by echocardiography, which detected CAA at the left main coronary trunk (2.3 mm, Z score = 3.2) and left circumflex coronary artery (1.8 mm, Z score = 2.8). Since KD in this case was refractory to the administration of IVIG, the patient was treated with aspirin and three courses of IVIG (2 g/kg/day on days 14, 19, and 35) plus 5 mg of cyclosporine from day 35 to 53. He was discharged on day 45 with small aneurysms present at the left main coronary trunk and the left circumflex coronary arteries. Left main coronary trunk was 2.4 mm (Z score = 2.2) and left circumflex coronary artery was 1.5 mm (Z score = 0.8) at 7 months after discharge.

In order to identify the causative agent of severe pneumonia that required mechanical ventilation, multiplex real-time PCR was carried out on tracheal aspirate and serum sample to detect the genomes of 163 viruses (47 DNA viruses and 116 RNA viruses), 68 bacterial species, and nine fungal species [10, 11]. Moreover, specific reverse-transcription PCR (RT-PCR) was performed to detect human parechovirus in serum and cerebrospinal fluid [12, 13]. DNA and RNA were extracted from the patient’s serum and tracheal aspirate using a Maxwell RSC Viral Total Nucleic Acid Purification Kit (Promega, Madison, WI). No infectious pathogens were detected in these samples collected at the time of hospitalization.

In addition, the patient’s serum cytokine profile demonstrated the following (normal values are shown in parentheses): interleukin (IL)-18, 175 pg/mL (< 500 pg/mL); IL-6, 410 pg/mL (< 5 pg/mL); neopterin, 36 nmol/L (< 5 nmol/L); soluble tumor necrosis factor receptor (sTNF-R)I, 3000 pg/mL (484–1407 pg/mL); and sTNF-RII, 14100 pg/mL (829–2262 pg/mL) by commercial ELISA (IL-18: MBL, Nagoya, Japan; IL-6, sTNF-RI, and sTNF-RII: R&D Systems, Minneapolis, MN, USA; neopterin: IBL, Hamburg, Germany) [14].

## Discussion and conclusion

In this case, two unusual clinical features are considered to have made it difficult to diagnose KD: the patient’s young age and the presence of severe pneumonia without typical clinical manifestations of KD. Because this neonatal patient had only fever, mild skin rash, and severe pneumonia at the time of admission, antibacterial and antiviral therapy was initiated empirically. The next day, his pneumonia deteriorated rapidly with the appearance of apnea and consolidation on chest CT. In addition to the rapid diagnostic tests for respiratory syncytial virus and human metapneumovirus at the time of admission, RT-PCR was performed retrospectively to detect human parechovirus because of fever, rash and apnea in the neonate [13], but it was negative. Although the patient’s condition gradually improved with intensive ICU treatment for 13 days, persistent leukocytosis and elevated levels of CRP were unusual. Typical clinical features of KD appeared 14 days after illness onset, and the patient was finally diagnosed with KD and treated appropriately.

Respiratory symptoms are rare in KD patients, although there have been several reports of KD with concurrent pneumonia [5, 7, 8, 15–17]. The clinical course of these patients was consistent with that in the present case in that the patients initially developed pneumonia and only subsequently demonstrated the characteristic symptoms of KD. However, these previously reported patients were 2 to 19 years of age, which is older than typical KD patients, and they did not require mechanical ventilation. Meanwhile, the present case was a neonate and had severe pneumonia that required mechanical ventilation. Therefore, it was important to determine whether this pneumonia was actually associated with KD. Multiplex real-time PCR to detect various respiratory pathogens and specific RT-PCR to detect human parechovirus were carried out retrospectively, but no pathogens were detected in any of the patient’s clinical specimens [10–13]. Monitoring cytokine profile is useful for differentiating diseases such as systemic-onset juvenile idiopathic arthritis and KD [14]. In this case, serum cytokine profile analysis performed on hospital admission revealed elevation of various cytokines, predominantly IL-6, corresponding to a typical, previously reported KD profile [14]. Therefore, the patient’s entire clinical course, including the severe pneumonia, was considered to represent KD. This is the first report of a neonatal KD patient with severe pneumonia requiring mechanical ventilation.

The pulmonary involvement in KD might be due to increased vascular permeability as occurs in other vasculitides. In the previous study, the expression of vascular endothelial growth factor was upregulated in blood vessels and many organs of patients who died of KD [18]. This may increase vascular permeability in various organs including lungs in severe KD patients, leading to edema and inflammation.

Although neonatal KD patients are extremely rare, there have been several case reports to date [19–25]. Two patients with apnea and two with pneumonitis have been reported, but in contrast to our case, these patients did not require mechanical ventilation to treat respiratory complications. In nationwide surveys in Japan, neonates accounted for 1/5,500 (0.018%) cases of KD. The clinical features of neonatal KD were incompatible with classic KD criteria, and the incidence of coronary artery lesions in neonatal KD patients was not statistically higher than that in older patients [21]. Another study also demonstrated that KD patients younger than 6 months were less likely to meet all the criteria for KD [3]. A literature review suggested that the incidences of fever (88%), skin rash (81%), and extremity changes (81%) were relatively high even in neonatal patients; however, the incidence of conjunctivitis was only 31% and that of cervical lymphadenopathy was 0% [19]. Consistent with this, the patient in this report had only fever and skin rash on hospital admission. In addition, an approximately 2-week interval was required to definitively diagnose KD based on the appearance of additional clinical manifestations, including fever recurrence, conjunctivitis, and extremity change. As already suggested, atypical clinical courses of this kind can cause delays in the accurate diagnosis of KD, leading to late treatment and poor prognosis. Many of these patients are suspected of having sepsis and are treated with antibiotics [19, 22, 23]. Therefore, if a febrile neonate does not respond to antimicrobial treatment, physicians should consider KD in the differential diagnosis.

As described above, infants with KD who have atypical clinical manifestations have been suggested to be at increased risk of coronary artery abnormalities [4]; fortunately, however, our patient had only mild CAA. This patient was administered IVIG soon after ICU admission to treat severe infectious disease. Although the IVIG dose was lower than that used to treat KD, it may have affected the clinical course, given the 2-week interval between disease onset and the appearance of typical KD symptoms and mild CAA despite delays in appropriate treatment. Therefore, reliable biomarkers for KD are needed to improve the prognosis of infants with KD who have an atypical clinical course. In the present case, a multiplex panel PCR test was retrospectively performed to identify causative agents of severe pneumonia, along with a cytokine profile analysis. However, these analyses are not appropriate for point-of-care testing of KD. It has been demonstrated that serum levels of lipopolysaccharide-binding protein, leucine-rich alpha-2-glycoprotein, angiotensinogen, and retinol-binding protein 4 may be predictive biomarkers of KD [26]. A prospective study to evaluate the reliability of these biomarkers is now underway in Japan.

## Data Availability

Not applicable.

## Abbreviations

CAA: Coronary artery aneurysms
CT: Computerized tomography
ICU: Intensive care unit
IL: Interleukin
IVIG: Intravenous immunoglobulin
KD: Kawasaki disease
RT-PCR: Reverse-transcription PCR
sTNFR: Soluble tumor necrosis factor receptor
